# Association of baseline as well as change in lipid levels with the risk of cardiovascular diseases and all-cause deaths

**DOI:** 10.1038/s41598-021-86336-6

**Published:** 2021-04-01

**Authors:** Hsin-Yin Hsu, Ming-Chieh Tsai, Tzu-Lin Yeh, Le-Yin Hsu, Lee-Ching Hwang, Kuo-Liong Chien

**Affiliations:** 1 Department of Family Medicine, Taipei MacKay Memorial Hospital, No. 92, Section 2, Zhongshan North Road, Taipei City, 10449 Taiwan; 2 Institute of Epidemiology and Preventive Medicine, National Taiwan University, Room 517, No. 17, Xu-Zhou Rd., Taipei City, 10055 Taiwan; 3 Department of Endocrinology, Department of Internal Medicine, Mackay Memorial Hospital, Tamsui Branch, Taipei City, 25160 Taiwan; 4 Department of Family Medicine, Hsinchu MacKay Memorial Hospital, Section 2, Guangfu Road, No. 690 Hsinchu City, 30071 Taiwan; 5 Department of Medicine, MacKay Medical College, No. 46, Sec. 3, Zhongzheng Rd, New Taipei City, 25245 Taiwan; 6 Department of Internal Medicine, National Taiwan University Hospital, No. 7, Zhongshan S. Rd., Zhongzheng Dist., Taipei City, 10002 Taiwan

**Keywords:** Cardiology, Risk factors

## Abstract

High baseline atherogenic lipid level has been an established risk factor for the risk of cardiovascular events. Evidence concerning the role of lipid changes in cardiovascular and death risks are inconclusive. A cohort study was conducted based on the Taiwanese Survey on Hypertension, Hyperglycemia, and Hyperlipidemia (n = 4072, mean 44.8 years, 53.5% women) assessing lipid levels of the participants repeatedly measured in 2002 and 2007. Combined baseline and changes in lipid levels were classified into four groups—stable or decreasing lipid changes and increasing lipid changes with low- and high-risk baseline lipid levels. Developing cardiovascular events (n = 225) and all-cause deaths (n = 345) were ascertained during a median follow-up of 13.3 years. Participants with increasing and higher total cholesterol level were more likely to develop cardiovascular risks. Similar patterns for cardiovascular events were observed across other lipid profile changes. However, participants with increasing total cholesterol, LDL-C, and non-high-density lipoprotein cholesterol (non-HDL-C) levels were more likely to be at a lower risk for all-cause deaths. Baseline and changes in total cholesterol, triglycerides, and LDL-C levels were positively associated with the risk of cardiovascular diseases, whereas baseline and changes in total cholesterol and LDL-C and non-HDL-C levels were inversely associated with all-cause deaths.

## Introduction

Cardiovascular disease (CVD) has long been the primary leading cause of death globally, responsible for almost one-third of all deaths each year^1,2^. Hyperlipidemia has been an important risk factor for CVD, especially high level of LDL-C. LDL-C has been the primary lipid target for prevention of CVD. However, previous studies have demonstrated that CVD events remain prevalent among individuals with low or normal LDL-C level, both pretreatment and during high-intensity lipid lowering therapy^3,4^. The significant residual cardiovascular risks were postulated mainly from inflammatory burden, atherogenic (apo)lipoproteins beyond LDL-C, such as apolipoprotein B (apo B), or non-HDL-C (calculated as the difference between total cholesterol and HDL-C levels)^5,6^.

High baseline atherogenic lipid levels have been an established risk factor for cardiovascular events, including ischemic coronary heart disease and ischemic stroke^7–12^. However, evidence concerning the role of lipid change for cardiovascular disease and death risks have been inconclusive. Only a few studies have revealed the association between the changes in lipid profile with CVD incidences^13–15^. Additionally, evidence regarding the association between baseline and the changes in lipid levels and all-cause death risks have remained controversial. In statin trials, intensive lipid lowering was associated with lower risk of all-cause deaths, while observational studies revealed that low total cholesterol was associated with a higher mortality risk^16–21^.

Thus, we conducted a prospective study to investigate if the baseline and changes in various lipid levels provide added predictive value for CVD, including coronary heart disease and ischemic stroke, and all-cause deaths. The second aim of our study was to explore crucial effect modifiers between lipoproteins and CVD, including coronary heart disease and ischemic stroke, and all-cause deaths^13,22^.

## Methods

### Study design and population

In this population-based prospective cohort study, the population was obtained from the 2002 Taiwanese Survey on Prevalence of Hypertension, Hyperglycemia, and Hyperlipidemia (TwSHHH) database, with follow-up in 2007^23^. The follow-up period ended when the participant developed cardiovascular disease, either coronary heart disease or ischemic stroke or all-cause death or lived beyond December 31, 2015.

This study was conducted according to the guidelines laid down in the Declaration of Helsinki. The protocol of our study was approved by the Institutional Review Board of National Taiwan University Hospital (NTUH-REC Number: 201901103W). TwSHHH data has followed the Institutional Review Board regulation, and informed consent has been collected accordingly.

In this study, individuals aged < 20 years or have been diagnosed with an evident CVD prior to TwSHHH 2007 were excluded. Individuals who were pregnant within 1 year of TwSHHH 2002 or 2007^24^ and had missing lipid profile data were also excluded.

This study used a joint TwSHHH database composed of five databases, namely, the 2001 National Health Interview Survey (NHIS 2001), TwSHHH 2002, TwSHHH 2007, the National Health Insurance Research Database (NHIRD), and the National Death Registry. TwSHHH 2002 was based on a sub-cohort randomly obtained from NHIS 2001 between March 2002 and October 2002. TwSHHH 2002 was the second nationwide health survey designed for national population samples. In TwSHHH 2002, all participants were interviewed with a questionnaire including complete health-related characteristics. In addition, participants had undergone physical examinations and laboratory biochemistry tests^23,25^. The follow-up survey of TwSHHH 2002 was conducted from June 2007 to May 2008. The Taiwan National Health Interview Survey has monitored the health of the nation periodically since 2001, collecting information of health topics through interviews. NHIS 2001 was the first general survey conducted from August 2001 to January 2002, using a stratified multistage systematic sampling procedure from 6592 households. The NHIRD contained data on the utilization of all NHI resources, including outpatient visits, hospital care, and prescribed medications. The TwSHHH database comprised data of populations in TwSHHH 2002 and 2007 linked with NIHS 2001, NHIRD, and National Death Registry.

### Data collection and measurements

Blood sampling was performed after a 12-h fasting period. The LDL-C level was measured using the Friedewald equation, i.e., “total cholesterol − HDL-C − (triglyceride/5),” in TwSHHH 2002 and by direct measurement with homogeneous assays in TwSHHH 2007. Total cholesterol and triglyceride levels were examined by colorimetry (Bucolo method), and HDL-C was examined by electrophoresis. Apo B and hsCRP levels were measured by immunoturbidimetric methods. The coefficients of variations of these laboratory measurements were approximately 5%. A standard protocol officially registered by the Taiwan Health Promotion Administration, Ministry of Health and Welfare was applied for health examinations and data collection.

### Definition of exposures

All lipid profile levels, including total cholesterol, triglyceride, LDL-C, non-HDL-C, and apo B, in TwSHHH 2002 were divided into quartiles. For the change value in lipid profiles between TwSHHH 2002 and 2007, they were divided into increasing, stable, and decreasing groups. Exposure was assessed by a two-step combination. In step 1, the baseline lipid levels were divided by quartiles. In step 2, baseline and change in lipid levels were combined. For baseline lipid levels, low-risk groups were defined as the lipid levels in the 1st and 2nd quartiles of the atherogenic (apo)lipoproteins, while the high-risk groups were defined as lipid levels in the 3rd and 4th quartiles of these lipids.

Although several variability indicators are commonly used to describe the changes in biochemistry data, such as standard deviation, variability independent of the mean, maximum–minimum difference, and average real variability, we chose standard deviation to present the change in lipid level due to following three reasons. First, standard deviation and variability independent of the mean are indicators to describe total variability of biochemistry data; however, standard deviation is generally known and easily computed. Second, standard deviation is suitable to describe data variability with different time intervals, either short term or long term, whereas average real variability is more appropriate for short-term variability and requires multiple measurement of lipid levels. Third, compared with maximum–minimum difference, standard deviation is less likely to be influenced by extreme observations^26–28^. For the change in lipid profiles, stable lipid change was defined as lipid change between TwSHHH 2002 and 2007 within ± 0.25 standard deviation of the baseline lipid level; decreasing lipid change was defined as lipid changes between TwSHHH 2002 and 2007 with 0.25 standard deviation of the baseline lipid profile or lower. Increasing lipid change was defined as lipid changes between TwSHHH 2002 and 2007 with 0.25 standard deviation of baseline lipid profile or higher. Then, four groups of combined lipid change were defined as follows: (1) low-risk group with stable or decreasing lipid levels, (2) low-risk group with increasing lipid levels, (3) high-risk group with stable or decreasing lipid levels, and (4) high-risk group with increasing lipid levels.

### Definition of outcomes

Each participant in the TwSHHH database was linked to the NHIRD to determine their outcomes. The primary endpoint was the incidence of CVD, including, coronary heart disease and ischemic stroke. The secondary endpoint was all-cause death. The outcomes of coronary heart disease and ischemic stroke were ascertained in NHIRD database by using the International Classification of Diseases, Ninth Revision-Clinical Modification (ICD9-CM) codes. Coronary heart diseases were recognized as at least one discharge diagnosis of ICD9-CM codes, including 410-413, 41400, 41401, 4148, 41489, v4581, v4582, and I20-I25, or procedure codes, including percutaneous coronary intervention (33076B, 33077B, and 33078B) and coronary bypass surgery (68023B, 68024B, 68025B, N26002, and N26003). Ischemic strokes or transient ischemic accidents were recognized as at least one discharge diagnosis of ICD-9-CM codes, including 433,434,435,436,437.1, 437.8, and 437.9. All-cause death was confirmed by the National Death Registry.

### Definitions of covariates

Significant covariates including age, sex, BMI, parental history of CVD, menopause status, smoking status, exercise, alcohol use, educational level, income level, and marital status were collected by questionnaires. Hypertension was defined as an average systolic blood pressure (SBP) of ≥ 140 mmHg or an average diastolic blood pressure (DBP) of ≥ 90 mmHg or use of antihypertensive medications more than 84 days within 1 year before the index day (JNC-7). Diabetes was defined as a fasting plasma glucose of ≥ 126 mg/dL or a hemoglobin A1c of ≥ 6.5% or use of antidiabetic agents more than 84 days within 1 year before the index day (Table S1)^29^.

### Statistical analyses

Relationships between baseline lipid marker levels were examined by the Pearson correlation coefficients. The correlation coefficients between the baseline lipid level and change in lipid levels was calibrated using Oldham’s method^30^. The Cox proportional-hazards regression was used to compare the hazard ratios (HRs) for the outcomes after the proportionality assumption was verified^31^. Potential confounders were adjusted via three models: model 1, adjusted for age group and sex; model 2, adjusted for factors in model 1 plus BMI, smoking status, alcohol use, regular exercise, income level, and educational level; and model 3, adjusted for factors in model 2 plus diabetes, hypertension, menopause, family history of CVD, and hsCRP level. Subgroup analyses were done to explore potential effect modifiers stratified by age, sex, LDL-C targeted or not, and median hsCRP. Sensitivity analyses were also performed to judge the robustness of our results: (1) excluding events within 1 year after TwSHHH 2007 in case of reverse causality; (2) excluding participants with triglycerides level ≥ 400 mg/dL in the TwSHHH 2002 because the LDL-C value in TwSHHH 2002 may be biased when obtained using the Friedewald formula if the triglyceride level was ≥ 400 mg/dL^32^; and (3) defining stable lipid change as lipid change between TwSHHH 2002 and 2007 within ± 0.5 standard deviation of the baseline lipid profile.

The level of statistical significance was set at a two-tailed alpha level < 0.05. Analyses were performed with SAS version 9.4 (SAS Institute, Cary, NC) and Stata version 14 (Stata Corporation, College Station, Texas).

## Results

**Figure 1:**
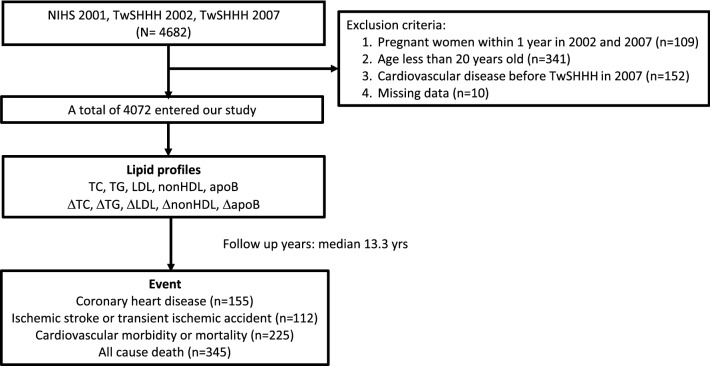
Flow chart of the study. *NIHS* National Health Interview Survey; *TwSHHH* Taiwanese Survey on Hypertension, Hyperglycemia, and Hyperlipidemia; *TC* total cholesterol; *TG* triglycerides; *LDL-C* low-density lipoprotein cholesterol; *non-HDL-C* non-high-density lipoprotein cholesterol; *ApoB* apolipoprotein B.

The study population included 4072 participants free of CVD who had data on lipid profiles at both baseline and follow-up surveys. The population’s mean (SD) age was 44.8 (14.9) years, and 53.5% were women. In total, 612 participants were excluded, among them were 109 participants who were pregnant within 1 year in TwSHHH 2002 and 2007, 341 who were < 20 years old, 10 who had missing data, and 152 who had established CVD before TwSHHH in 2007. The study flow chart is shown in Fig. 1. During a median follow-up of 13.3 years for CVD, coronary heart disease, and ischemic stroke and 13.4 years for all-cause death, we documented 252 cases of incident CVD, 155 of incident coronary heart events, 112 of incident ischemic strokes, and 345 of all-cause deaths.

Baseline characteristics of the study population in each non-HDL-C quartile are displayed in Table 1. Participants with higher non-HDL-C levels tended to be men, older, and heavier. Hypertension, diabetes mellitus, menopause, current smokers, high monthly income level, family history of CVD, and failure of LDL-C target achievement were more common in the higher non-HDL-C quartiles. Higher non-HDL-C quartiles were positively associated with higher levels of total cholesterol, triglycerides, LDL-C, apo B, and hsCRP. Lifestyle factors such as alcohol consumption and exercise habits did not vary across various non-HDL quartiles. The range and median value of baseline lipid level specified by combined lipid change are demonstrated in Table S2.1 and S2.2. We found strong correlations between baseline total cholesterol, triglycerides, LDL-C, non-HDL-C, and apo B (correlation coefficients ranging from 0.78 to 0.91). In addition to correlation among total cholesterol and LDL-C, non-HDL-C was higher than that with apo B, both in the baseline lipid levels and lipid change (Table S3).

**Table 1:** Distribution of various baseline demographic, lifestyle, and socioeconomic factors in the study population, specified by NonHDL quartiles.

	1	2	3	4	*p*
Number	922	1030	1083	1035	
Range (mg/dL)	< 93	105–124	125–148	> 149	
Median (mg/dL)	54	114	135	168	
Women	557(60.4)	583(56.6)	562(51.9)	474(45.8)	< .0001
**Age (years)**					< .0001
20–64	862(93.5)	922(89.5)	932(86.1)	868(83.9)	
≥ 65	60(6.5)	108(10.5)	151(13.9)	167(16.1)	
**Body mass index** (**kg**/**m**^2^)					< .0001
< 18.5	82(10.7)	63(7.3)	37(4.2)	14(1.6)	
18.5–23.9	509(66.5)	536(62)	462(51.9)	341(40)	
24.0–-26.9	122(15.9)	183(21.2)	263(29.6)	294(34.5)	
≥ 27.0	53(6.9)	82(9.5)	128(14.4)	203(23.8)	
Diabetes mellitus	44(4.8)	51(5.0)	100(9.2)	159(15.4)	< .0001
Hypertension	80(8.7)	95(9.2)	208(19.2)	258(24.9)	< .0001
Menopause	84(9.1)	148(14.4)	251(23.2)	269(26)	< .0001
Current smoker (yes)	154(16.7)	209(20.3)	198(18.3)	248(24)	0.000
Alcohol drinking (yes)	205(22.2)	249(24.2)	253(23.4)	266(25.7)	0.33
Regular exercise (yes)	211(22.9)	225(21.8)	266(24.6)	240(23.2)	0.52
**Marital status**					< .0001
Single, divorced or separated	453(49.1)	414(40.2)	351(32.4)	317(30.6)	
Living with spouse	469(50.9)	616(59.8)	732(67.6)	718(69.4)	
**Education level**					< .0001
< 9 years	395(42.8)	480(46.6)	599(55.3)	612(59.1)	
≥ 9 years	527(57.2)	550(53.4)	484(44.7)	423(40.9)	
Low income level	683(74.1)	735(71.4)	743(68.6)	724(70)	0.05
Family history of CVD	168(18.2)	238(23.1)	254(23.5)	279(27)	< .0001
Target achieved of LDL-C	922(100)	1016(98.6)	992(91.6)	558(53.9)	< .0001

Variable	Mean (SD)	Mean (SD)	Mean (SD)	Mean (SD)	*p*
Age(years)	38.2(14.2)	43(14.8)	47.7(14.4)	49.5(13.7)	< .0001
Body mass index(kg/m^2^)	22.0(3.2)	22.7(3.3)	23.8(3.4)	24.9(3.7)	< .0001
Lipid profiles(mg/dL)					
Total cholesterol	145(16.5)	171.8(14.7)	192.2(16.9)	229.1(33)	< .0001
Triglycerides	83.0(40.1)	102.5(46.0)	129.7(65.0)	198.4(115.1)	< .0001
LDL cholesterol	85.7(9.2)	106.3(7.5)	123.2(9.4)	148.7(23.7)	< .0001
HDL cholesterol	53.6(12.1)	57.4(13.6)	57.0(15.4)	53.8(18.5)	< .0001
NonHDL cholesterol	91.4(9.5)	114.4(5.6)	135.1(6.9)	175.3(28.9)	< .0001
Apolipoprotein B	64.7(12.5)	80.3(12)	96(13.5)	117.1(21.4)	< .0001
hsCRP	0.16(0.39)	0.2(0.43)	0.25(0.69)	0.31(0.78)	< .0001
∆ Total cholesterol	9.1(27.6)	0.8(26)	− 0.8(29.4)	− 21(39)	< .0001
∆ Triglycerides	8.3(49)	4.2(53.3)	1.9(77.4)	− 22.5(109.9)	< .0001
∆ LDL cholesterol	3.0(22.8)	− 0.8(24)	− 1.9(27.7)	− 17.1(35.3)	< .0001
∆ HDL cholesterol	− 0.5(12.6)	− 4.3(12.1)	− 4.7(14)	− 3.4(16.6)	< .0001
∆ nonHDL cholesterol	9.5(24.1)	5.1(24.4)	3.9(27.4)	− 17.6(37.6)	< .0001
∆ apolipoprotein B	10.3(12.9)	6.3(13.7)	2.9(15.6)	− 7(20.7)	< .0001

*CVD* cardiovascular disease; *LDL-C* low-density lipoprotein cholesterol; *HDL cholesterol* high density lipoprotein cholesterol; *NonHDL cholesterol* non-high density lipoprotein cholesterol; To convert total cholesterol, LDL cholesterol, HDL cholesterol, nonHDL cholesterol and apolipoprotein B from mg/dl to mmol, divide by 38.46; To covert triglycerides from mg/dl to mmol , divide by 87.72.

Proportional hazard assumption was verified in the Cox models. Table 2 shows the HRs and 95% confidence intervals (CIs) for CVD according to various lipid profiles at baseline. After adjustment for potential confounders, the HR for the comparison of the participants was 1.70 (95% CI, 1.00–2.89; P for trend, 0.07) in the fourth and first total cholesterol quartiles, 1.21 (95% CI, 0.70–2.10; P for trend, 0.36) in the fourth and first triglyceride quartiles, 1.51 (95% CI, 0.89–2.59; P for trend, 0.16) in the fourth and first LDL-C quartiles, 1.70 (95% CI, 0.94–3.09; P for trend, 0.17) in the fourth and first non-HDL-C quartiles, and 2.05 (95% CI, 1.03–4.08; P for trend, 0.08) in the fourth and first apo B quartiles. Similar patterns for coronary heart disease and ischemic stroke were observed. However, no relationships between baseline lipid profile and risk of all-cause deaths were observed (Table S4).

**Table 2:** Hazard ratios (and 95% CI values) of cardiovascular disease risk during a median 13.3 years of follow-up according to baseline lipid profiles in TwSHHH 2002.

	1	2	3	4	Trend test
**Total cholesterol**					
Events	24	49	61	91	
Person-years	12,541	14,046	13,632	13,716	
Incidence rate	1.9	3.5	4.5	6.6	
Model1	1	1.66(0.94–2.91)	1.80(1.03–3.14)	2.67(1.58–4.50)	< .0001
Model2	1	1.57(0.89–2.75)	1.53(0.87–2.67)	2.13(1.26–3.62)	0.004
Model3	1	1.45(0.83–2.55)	1.49(0.86–2.58)	1.70(1.00–2.89)	0.07
**Triglycerides**					
Events	23	38	70	91	
Person-years	12,870	13,220	13,882	13,607	
Incidence rate	1.8	2.9	5	6.7	
Model1	1	1.19(0.66–2.17)	1.96(1.14–3.37)	2.4(1.41–4.08)	0.000
Model2	1	1.08(0.59–1.97)	1.59(0.92–2.77)	1.82(1.05–3.17)	0.010
Model3	1	0.94(0.53–1.69)	1.27(0.74–2.17)	1.21(0.70–2.10)	0.36
**LDL cholesterol**					
Events	23	46	72	84	
Person-years	12,369	14,065	14,143	13,358	
Incidence rate	1.9	3.3	5.1	6.3	
Model1	1	1.33(0.75–2.36)	1.95(1.15–3.30)	2.16(1.29–3.64)	0.001
Model2	1	1.25(0.70–2.21)	1.60(0.94–2.72)	1.72(1.02–2.91)	0.029
Model3	1	1.34(0.76–2.38)	1.33(0.77–2.30)	1.51(0.89–2.59)	0.16
**NonHDL cholesterol**					
Events	20	43	68	94	
Person-years	12,393	13,708	14,339	13,495	
Incidence rate	1.6	3.1	4.7	7.0	
Model1	1	1.78(0.97–3.27)	2.25(1.26–4.01)	2.79(1.58–4.92)	0.000
Model2	1	1.58(0.86–2.91)	1.76(0.98–3.16)	2.08(1.17–3.69)	0.015
Model3	1	1.62(0.87–3.02)	1.51(0.83–2.77)	1.70(0.94–3.09)	0.17
**Apolipoprotein B**					
Events	16	41	70	98	
Person–years	12,058	14,040	13,994	13,801	
Incidence rate	1.3	2.9	5.0	7.1	
Model1	1	2.85(1.37–5.94)	2.97(1.44–6.13)	5.03(2.50–10.13)	< .0001
Model2	1	2.44(1.17–5.10)	2.27(1.09–4.71)	3.48(1.70–7.11)	0.001
Model3	1	1.80(0.89–3.64)	1.76(0.88–3.53)	2.05(1.03–4.08)	0.08

Incidence rates are presented per 1000 person-years; Model 1: adjusted for age groups (20–64/ ≥ 65 years old) and sex. Model 2: as for model 1 plus body mass index (18.4/18.5–23.9/24.0–26.9/ ≥ 27 kg/m2), current smoker (yes/no), alcohol drinking (yes/no), marital status (single, divorced or separated/Living with spouse), regular exercise habit (yes/no),education level (9 years/at least 9 years), income level (monthly income level < 40,000, ≥ 40,000 New Taiwan Dollars). Model 3: as for model 2 plus baseline hypertension (yes/no), diabetes mellitus (yes/no), menopause(yes/no), family history of CVD (yes/no), and hsCRP level.

**Figure 2:**
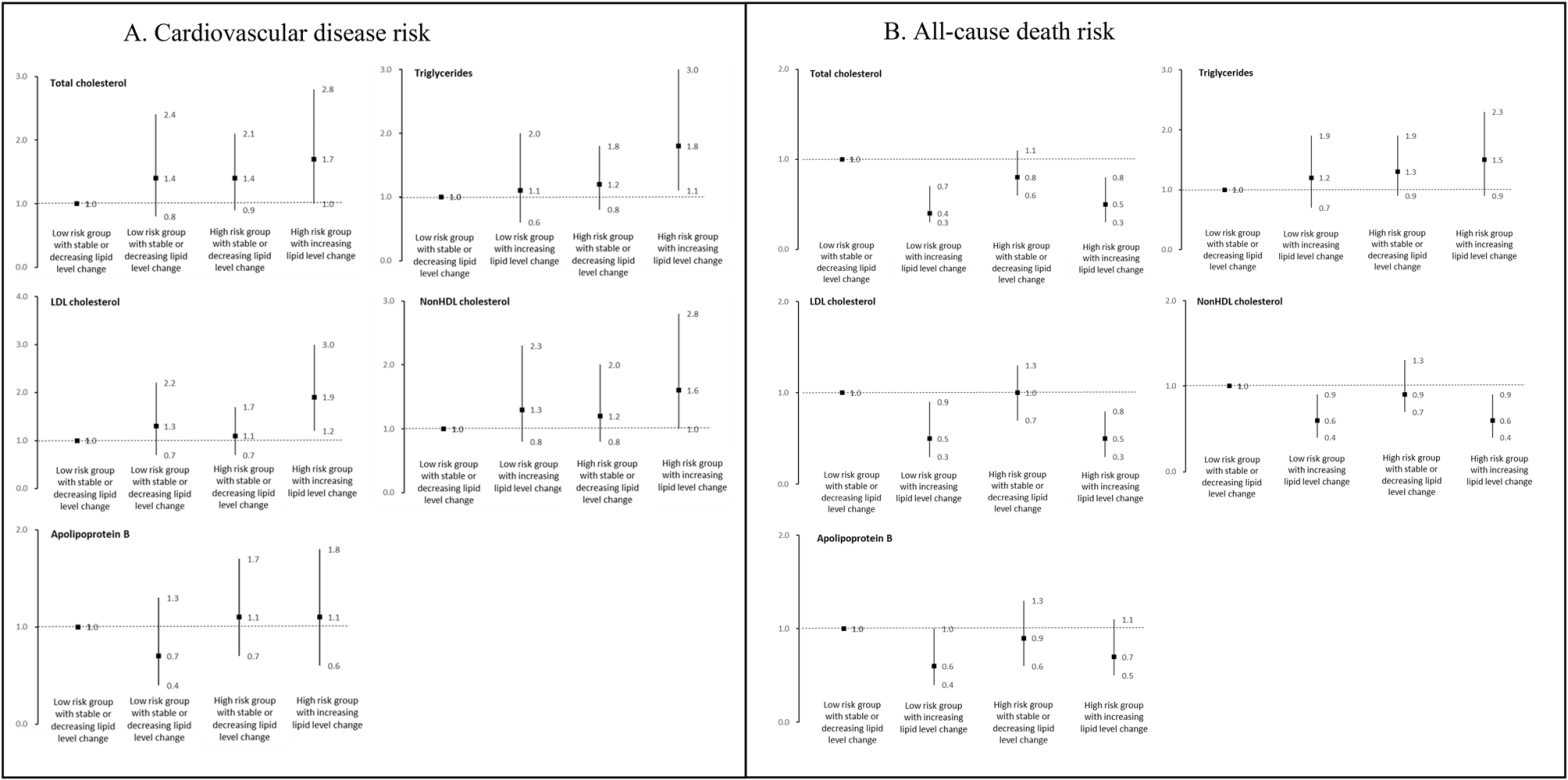
Joint effects of baseline lipid profile and the change of lipid change for cardiovascular disease and all-cause death risks. Hazard ratios were adjusted for age groups (20–64/ ≥ 65 years old), sex, body mass index (18.4/18.5–23.9/24.0–26.9/ ≥ 27 kg/m2), current smoker (yes/no), alcohol drinking (yes/no), marital status (single, divorced or separated/Living with spouse), regular exercise habit (yes/no),education level (9 years/at least 9 years), income level (monthly income level < 40,000, ≥ 40,000 New Taiwan Dollars), baseline hypertension (yes/no), diabetes mellitus (yes/no), menopause(yes/no), family history of CVD (yes/no), and hsCRP level.

Figure 2A shows the joint analyses of baseline and change in lipid levels for CVD risk. Compared with participants with decreasing and lower total cholesterol levels, those with increasing and higher total cholesterol levels were more likely to develop CVD (HR, 1.38, 95% CI, 0.81–2.35, for low total cholesterol levels; HR, 1.70, 95% CI, 1.01–2.84, for high total cholesterol levels). Similar patterns for cardiovascular events were found with triglycerides (HR, 1.09, 95% CI, 0.61–1.95, for low triglycerides levels; HR, 1.81, 95% CI, 1.11–2.97, for high triglycerides levels), LDL-C (HR, 1.27, 95% CI, 0.74–2.17, for low LDL-C levels; HR, 1.87, 95% CI, 1.15–3.04, for high LDL-C levels), and non-HDL-C (HR, 1.32, 95% CI, 0.75–2.32, for low non-HDL-C level; HR, 1.64, 95% CI, 0.97–2.77, for high non-HDL-C level) lipid change. However, increasing apo B levels were not associated with higher CVD risk compared with decreasing or stable apo B levels (HR, 0.74, 95% CI, 0.41–1.31, for low apo B level; HR, 1.07, 95% CI, 0.63–1.83, for high apo B level). Similar patterns for coronary heart disease and ischemic stroke were observed across other lipid change except apo B.

We found that increasing lipid change was inversely associated with all-cause death (Fig. 2B). Compared with participants with decreasing and lower non-HDL-C levels, those with increasing non-HDL-C levels were likely to have a lower risk for all-cause death (HR, 0.59, 95% CI, 0.37–0.94, for low non-HDL-C level; HR, 0.57, 95% CI, 0.36–0.90, for high non-HDL-C level). Similar patterns for all-cause death were observed across other lipid changes.

Subgroup analyses revealed that age may be an effect modifier for the association between baseline lipid levels and the risk of CVD (interaction p as 0.033 in non-HDL-C), indicating that elevated non-HDL-C levels had more pronounced effects on the risk of CVD in the younger population than the older population (Table 3). We found the association between baseline apo B levels and the risk of coronary heart disease varied by the status of LDL-C target achievement. This suggests that elevated apo B levels potentially play an essential role in the risk of coronary heart disease in the population who achieved their LDL-C targets (interaction p as 0.023 in apo B) (Table S5.1).

**Table 3:** Subgroup analyses for the associations of the levels of the lipoproteins (higher risk group vs. lower risk group) with incident cardiovascular disease according to age, sex, target achievement status of low-density lipoprotein and different levels of hsCRP.

Variable	Total cholesterol	*p*_interaction_	Triglycerides	*p*_interaction_	LDL cholesterol	*p*_interaction_	NonHDL cholesterol	*p*_**interaction**_	Apolipoprotein B	*p*_**interaction**_
**Age(years)**		0.49		0.40		0.07		0.033		0.18
20–64	1.22(0.79–1.87)		1.35(0.84–2.15)		1.36(0.88–2.11)		1.40(0.88–2.24)		1.28(0.80–2.05)	
≥ 65	1.26(0.75–2.12)		1.20(0.72–2.03)		0.95(0.57–1.57)		0.90(0.54–1.51)		1.08(0.64–1.82)	
**Sex**		0.78		0.26		0.35		0.61		0.35
Women	1.21(0.70–2.10)		1.65(0.94–2.88)		1.04(0.61–1.77)		1.09(0.62–1.90)		1.02(0.59–1.74)	
Men	1.35(0.89–2.05)		1.12(0.72–1.76)		1.32(0.86–2.04)		1.27(0.81–1.98)		1.41(0.88–2.25)	
**Target achieved of LDL–C**		0.67		0.68		0.72		0.95		0.14
Yes	1.27(0.86–1.88)		1.15(0.77–1.73)		1.10(0.74–1.64)		1.13(0.76–1.68)		1.32(0.88–1.99)	
No	1.93(0.65–5.73)		1.45(0.70–3.02)		1.74(0.52–5.82)		1.64(0.38–7.10)		0.66(0.28–1.56)	
**hsCRP level**		0.76		0.90		0.75		0.93		0.42
hsCRP < median	1.24(0.69–2.23)		1.14(0.65–2.02)		0.96(0.55–1.70)		1.04(0.58–1.88)		1.31(0.72–2.38)	
hsCRP ≥ median	1.27(0.85–1.89)		1.34(0.86–2.09)		1.28(0.84–1.96)		1.21(0.78–1.87)		1.15(0.74–1.77)	

*LDL-C* low density lipoprotein cholesterol.

The sensitivity analyses demonstrated that our results were consistent with those obtained in the main analyses (Table S6.1-S6.2, Table S7.1-S7.2).

## Discussion

Our study suggested a positive association between baseline lipid levels and the incidence of CVD, including coronary heart disease and ischemic stroke in Taiwanese populations. These findings were more pronounced in populations aged < 65 years. Higher baseline levels of apo B had a stronger risk of coronary heart diseases in the target achievement of LDL-C levels. Furthermore, the increasing change in lipid levels was significantly associated with increased future risk of CVD, including coronary heart disease and ischemic stroke. No significant association between baseline lipid level and the risk of all-cause death was found, while increasing lipid change was significantly inversely associated with all-cause death.

Previous studies have demonstrated the consistency of high baseline lipid levels with the risk of CVD, coronary heart disease, and ischemic stroke^6,9–11,33–35^. The relationship between (apo)lipoproteins with CVD may be distinct in different populations such as younger individuals, women, and populations who achieved their LDL-C target^11,22,35,36^. Our data consistently suggested that younger populations with higher baseline lipid levels had significantly greater risks of CVD. Furthermore, LDL-C achievement in our study consistently acted as an effect modifier between non-LDL (apo)liproteins and cardiovascular events.

While many studies have evaluated the association between baseline lipid levels and the risks of CVD and all-cause death, limited evidence elucidated the relation of the change in lipid levels to the risks of CVD and all-cause death^13–19,37,38^. In a cohort study including 2,682,045 Korean participants using category change according to the cholesterol levels between the two periods (sustained low, low-middle, low–high, middle-low, sustained middle, middle-high, high-low, high-middle, and sustained high groups) to describe cholesterol variability, the HRs for comparisons of the increasing and stable total cholesterol group were 1.21 (95% CI, 1.03–1.42, in the low–high group) for coronary heart disease and 1.24 (95% CI, 1.05–1.47, in low–high group) for cerebrovascular disease^13^. In the present study, we explored the association between cardiovascular risks and various atherogenic (apo)lipoproteins (both the baseline and the change in lipid levels), which provided considerable information of their roles in the atherogenesis in coronary heart disease and ischemic stroke.

A novel finding in our study was that increasing apo B level showed no higher cardiovascular risks, implying that apo B may play a different role in the formation and progression of atherosclerosis than did total cholesterol, LDL-C, and non-HDL-C.

The inconsistent results of studies elucidating the relationship between baseline lipid level and all-cause death risk may be attributed to the difference in age distribution. Older population tended to yield U-curve or inverse associations between baseline lipid levels and all-cause deaths^16–18,35^. The lack of association between baseline lipid level and all-cause death in our study may be due to a broad range of age distribution (20–93 years).

Epidemiological studies of lipid changes and all-cause death are limited and have yielded inconsistent findings^19,20,37^. In an 8.0-year follow-up cohort study of 269,391 Koreans, change in total cholesterol levels, especially in decreasing change from high baseline total cholesterol levels (3rd tertile to 1st tertile) had higher risk of all-cause death (HR, 1.47, 95% CI, 1.32–1.64) compared with stable middle total cholesterol levels^18^. However, in a meta-analysis of lipid-lowering trials, Cholesterol Treatment Trialists’ Collaboration reported that decreasing LDL levels reduced all-cause death risk (RR, 0.91, 95% CI, 0.88–0.93, per 1 mmol/L reduction in LDL-C)^20^. The inconsistency may occur because the populations in lipid-lowering trials had higher baseline cardiovascular risk, and their CVD-related deaths accounted more for all-cause death compared with that in population-based studies^4,39–45^.

Atherogenic (apo)lipoproteins act differently in atherogenesis^6^. Total cholesterol represents the total cholesterol mass in all lipoprotein particles. Non-HDL-C represents cholesterol mass of very-low-density lipoprotein (VLDL), intermediate density lipoprotein (IDL), and LDL particles. LDL-C is the cholesterol mass of LDL particles. Apo B reflects the total number of atherogenic lipoprotein particles because each of these lipoproteins contains one apo B molecule. Baseline apo B contributing more risk of cardiovascular events than the change in apo B level may indicate that the atherogenic risk is more closely related to the influx of cholesterol mass to the arterial wall than to the particle numbers^6,9,11,46,47^.

The positive association between decreasing lipid change and all-cause death may be explained by at least two potential mechanisms. First, the drop in lipid levels indicate reduced resistance to oxidative stress due to lipid and may play an essential role while cells are involved in tissue repair^17^. Second, a decrease in lipid levels may interfere with the binding of lipopolysaccharide from microorganisms, which increases the risks of infection^38^. Third, lipids play an essential role in cell stability. Decreasing lipid changes can disturb cell integrity and cell growth, which may increase the risk of immunological and hematological diseases and malignancy^35,38^. Lastly, the decline in lipid levels may be attributed to malnutrition or diseases in a subclinical stage, which further increases the risk of mortality^18,37^.

For primary prevention of CVD, lipid change is as important as baseline lipid levels in predicting the risks of CVD, including coronary heart disease and ischemic stroke. This finding reminds clinicians that monitoring baseline lipid levels and the trend of the lipid change is necessary^34,48^. To reduce these lipid profiles, early intervention should be implemented even in relatively low baseline lipid level and younger populations to prevent cardiovascular events. In addition to LDL-C, the established primary target to prevent CVD and the monitoring of other atherogenic (apo)lipoprotein levels cannot be ignored^13,34^. In populations that achieved the LDL-C target, lowering baseline apo B is beneficial and necessary to reduce the lipid-related residual cardiovascular risks^3,5^.

Furthermore, our study showed that those with decreasing lipid change had increased mortality risk. In case of decreasing lipid levels, awareness of potential preclinical nonatherosclerotic diseases to prevent further elevated all-cause death risk is strongly recommended for primary care providers.

### Strengths and limitations

This study has the following strengths. First, to our knowledge, this study is the first extensive investigation of baseline lipid level as well as lipid change and the risk of CVD, including coronary heart disease and ischemic stroke among the Taiwanese population. Evidence of single lipid profile change being positively associated with CVD risk was found in Asian populations, but no study has investigated combined baseline and change in lipid profiles in predicting CVD. Our study provided robust evidence by exploring the relations of various combinations of baseline lipid and lipid change level to the risks of CVD, including coronary heart disease and ischemic stroke, and all-cause death. Second, our study is a nationwide representative cohort study with ascertained outcomes based on the TwSHHH database. The community-based populations from the TwSHHH database may reduce the selection bias. The TwSHHH database included important clinical laboratory variables and socioeconomic and lifestyle factors to adjust for potential confounding factors. Finally, our study emphasized the importance of lipid-related residual cardiovascular risk.

Our study also has several potential limitations. First, this cohort study was restricted to the Taiwanese population; thus, our results lacked external generalizability. Second, relatively few incident cases of cardiovascular events were observed for risk estimations. However, a median 13.3-year follow-up made our results robust. Third, limited information elucidating the influence of medication use on CVD was due to the primary prevention study design. Fourth, apart from standard deviation, variability independent of the mean, maximum-minimum difference and average real variability are also indicators to describe the lipid variability. However, we have limited data measurement points currently. Future longitudinal data with enough data points of lipid level may be considered to elucidate the lipid variability more thoroughly.

In conclusion, this prospective study provided suggestive evidence on how baseline and changes in lipid profile were associated with the risks of cardiovascular diseases and all-cause death. Baseline and changes in total cholesterol, triglycerides, and LDL-C levels were positively associated with the risk of cardiovascular diseases, whereas baseline and changes in total cholesterol, LDL-C, and non-HDL-C levels were inversely associated with all-cause death. Further studies about biological mechanism for the role of baseline and change in lipid levels are warranted.

## Supplementary Information

Supplementary Information
