# Supplementary material for: Association of baseline as well as change in lipid levels with the risk of cardiovascular diseases and all-cause deaths
Source: Sci Rep. 2021 Apr 1;11:7381. doi: 10.1038/s41598-021-86336-6 (PMC8016969; doi:10.1038/s41598-021-86336-6)
Supplement: Supplementary file 1 — Supplementary Information [file 41598_2021_86336_MOESM1_ESM.docx]

**Association of Baseline as well as Change in Lipid Levels with the Risk of Cardiovascular Diseases and All-Cause Deaths**

*Short title: Lipid change and cardiovascular disease risk*

Hsin-Yin Hsu^1, 2^, Ming-Chieh Tsai^2,3^, Tzu-Lin Yeh^2,4^, Le-Yin Hsu^2^, Lee-Ching Hwang^1,5^, Kuo-Liong Chien^2,6^

^1^ Department of Family Medicine, Taipei MacKay Memorial Hospital, 10449, No. 92, Section 2, Zhongshan North Road, Taipei City, Taiwan 10449

^2^ Institute of Epidemiology and Preventive Medicine, National Taiwan University, Taipei City, Taiwan. Room 517, No. 17, Xu-Zhou Rd., Taipei City, Taiwan 10055

^3^ Department of Endocrinology, Department of Internal Medicine, Mackay Memorial Hospital, Tamsui Branch, Taiwan 25160

^4^ Department of Family Medicine, Hsinchu MacKay Memorial Hospital, No. 690, Section 2, Guangfu Road, Hsinchu City, Taiwan 30071

^5^ Department of Medicine, MacKay Medical College, No. 46, Sec. 3, Zhongzheng Rd. New Taipei City, Taiwan 25245

^6^ Department of Internal Medicine, National Taiwan University Hospital, No. 7, Zhongshan S. Rd., Zhongzheng Dist., Taipei City, Taiwan 10002

**Table S1 Drug codes in National Health Insurance Research Database to define medication use in the study population**

| Medication | Drug codes |
| --- | --- |
| Anti-hypertensive agents | AC36675100,BC16080100,BC20238100,AB43852100,AC46867100,BC21303100,A031032100,A036844100,AB41956100,AC29301100,AC29313100,BC16888100,BC20174100,BC23799100,A041679100,AB29478100,AB294781G0,AB32322100,AB323221G0,AB33029100,AB330291G0,AB34359100,AB343591G0,AB361291G0,AB36130100,AB361301G0,AB38867100,AB388671G0,AB44843100,AB448431G0,AB515971G0,AC27452100,AC274521G0,AC29443100,AC294431G0,AC29735100,AC297351G0,AC31223100,AC312231G0,AC33124100,AC331241G0,AC35367100,AC353671G0,AC3612910,AC37928100,AC379281G0,AC39816100,AC398161G0,AC39843100,AC398431G0,AC41497100,AC414971G0,AC43598100,AC435981G0,AC43704100,AC437041G0,AC44163100,BC22144100,BC22158100,BC221591G0,BC221601G0,A042909100,AB41669100,AB47472100,AC42967100,AC42999100,BC06049100,AB44619100,AC45354100,BC21530100,BC22553100,AB45348100,AB47538100,AB48439100,AB55406100,AB55984100,AC48905100,AC49083100,AC49504100,AC49544100,AC49623100,AC4965010,AC49851100,AC49915100,AC50061100,BC17090100,BC17125100,BC24039100,BC26700100,A046026100,A046140100,AC30712100,AC31226100,AC32350221,AC39703100,AC49266100,AC49550221,BC17560100,BC17586100,BC24532100,AC44731100,AC46404100,AC46699100,AC47239100,AC47295100,AC47434100,AC47460100,AC47510100,AC47894100,AC48168100,AC48309100,AC49358100,AC49469100,AC55987100,AC57128100,AC57370100,BC20452100,BC22071100,BC24967100,BC25070100,BC25674100,AC29804100,AC34300100,A019039100,A020613100,A0214801G0,A0384871G0,AC14079100,AC14981209,AC21480100,AC21684100,AC23764100,AC26144100,AC38487100,BC25391100,AA49920100,AA57838100,AB44621100,AC26741100,AC267411G0,AC30415100,AC39962100,AC42378100,AC423781G0,AC42874100,AC43467100,AC43765100,AC44567100,AC44853100,AC448531G0,AC45013100,AC45039100,AC450391G0,AC45144100,AC50263100,AC57288100,AC57929100,AC59902100,AC599021G0,BC216411G0,BC216431G0,BC23179100,BC23711100,A005266100,A012898100,A025245209,A025245277,AC00178100,AC001781G0,AC02756100,AC07342100,AC18232100,AC30742100,AC30743100,AC30879100,AC31534100,AC315341G0,AC31540100,AC315401G0,AC32117100,AC321171G0,AC32851100,BC07538100,BC21879248,A016964100,A030456100,A031179100,AB03388100,AB033881G0,AC03098100,AC030981G0,AC04276100,AC042761G0,AC10917100,AC109171G0,AC14346100,AC40294100,AC46028100,AC460281G0,AC58612100,AC586121G0,N003316100,NC00141100,NC001411G0,NC001411GA,NC02597100,NC02791100,NC027911G0,NC05874100,NC08138100,NC081381G0,NC11948100,NC16648100,NC166481G0,A026027100,A027818100,A028200100,A046691100,A047171100,A047420100,A051388100,AB41641100,AB45955100,AB47372100,AC34090100,AC42380100,AC423801G0,AC42659100,AC42749100,AC43728100,AC44828100,AC46538100,AC473721G0,AC47606100,AC47789100,AC47996100,AC48122100,AC48705100,AC487051G0,AC50136100,AC50254100,AC50772100,BC16528100,BC21754100,BC223871G0,A0342831G0,AB27928212,AB30749100,AB307491G0,AB44046157,AB45363100,AB453631G0,AC01047100,AC010471G0,AC02368100,AC023681G0,AC11150212,AC22641100,AC226411G0,AC25164219,AC26154100,AC27244100,AC27577212,AC28079100,AC30521212,AC30697100,AC306971G0,AC14015100,AC18569100,AC185691G0,AC22908100,AC229081G0,AC25965100,AC259651G0,AC30526100,AC305261G0,AC41545100,AC415451G0,AC50116100,AC501161G0,AC501161G4,AC501161G5,AC501161G6,AC59370100,BC22610100,BC226101G0,BC24306100,BC27122100,BC27255100,A049307100,AA42704100,AA45670100,AB44098100,AB44468100,AB44475100,AB44607100,AB45392100,AB46480100,AB49969100,AB58075100,AB58278100,AC24018100,AC27439100,AC28131100,AC29436100,AC294361G0,AC32801100,AC328011G0,AC34553100,AC35891100,AC38154100,AC38996100,AC40168100,AC401681G0,AC41058100,AC42840100,AC42857100,AC42917100,AC43931100,AC43970100,AC44371100,AC44575100,AC44631100,AC44687100,AC44882100,AC44925100,AC45085100,AC45177100,AC45187100,AC45448100,AC45510100,AC46046100,AC47093100,AC47536100,AC47626248,AC47632100,AC48064100,AC48313100,AC48465100,AC48559100,AC48600100,AC48803212,AC48803229,AC48803240,AC48833100,AC48859100,AC48882229,AC48994100,AC49057100,AC49376100,AC49527100,AC49876100,AC49963100,AC49966100,AC50138100,AC52520100,AC52616100,AC55540100,AC55552100,AC55896100,AC56633100,AC56681100,AC56716100,AC56780100,AC57114100,AC57219100,AC57368100,AC58208100,AC58310100,AC58993229,AC58993238,AC59056100,AC59415100,AC59781263,AC60161212,BA21344100,BA22951100,BB24898100,BC17469100,BC17488100,BC18842100,BC18843248,BC19856100,BC198561G0,BC19884100,BC20021229,BC20254100,BC21149100,BC21571100,BC225931G0,BC23293100,BC24364100,BC25198100,BC25199100,BC26222229,BC26274100,BC26415100,BC27093100,BC27139100,A0398791G0,AC27307212,AC27775100,AC277751G0,AC28032100,AC30120100,AC301201G0,AC30615100,AC306151G0,AC31537100,AC315371G0,AC33822100,AC37229100,AC38926100,AC39879100,AC43071100,BC16350212,BC17118100,BC17292100,BC19953100,BC23708100,BC237081G0,AB31889100,AB38600100,AC10358100,AC103581G0,AC30629100,AC306291G0AC32876100,AC33224100,AC332241G0,AC34373100,AC35996100,AC359961G0,AC36966100,AC369661G0,AC38422100,AC45926100,AC46439100AC48127100,AC48128100,AC57887100,BC18540248,BC19885100,BC22678100,BC22997100,BC23001100,A036790100,A040779100,A040949100,A041281100,A042346100,A042711100,A042712100,A044315100,A044810100,AB36788100,AB367881G0,AB48836100,AB51029100,AB54966100,AC30452100,AC304521G0,AC32275100,AC322751G0,AC32706100,AC32711100,AC33910100,AC33915100,AC34598100,AC345981G0,AC34880100,AC348801G0,AC35420100,AC354201G0,AC35836100,AC37204100,AC37833100,AC37835100,AC38204100,AC382041G0,AC39401100,AC394011G0,AC39414100,AC394141G0,AC39974100,AC399741G0,AC41430100,AC42584100,AC425841G0,AC42824100,AC43210100,AC43330100,AC43950100,AC43951100,AC44161100,AC443151G0,AC44420100,AC444201G0,AC444201G2,AC44477100,AC44480100,AC444801G0,AC448101G0,AC45172100,AC45254100,AC452541G0,AC45975100,AC46199100,AC46995100,AC47002100,AC48119100,AC481191G0,AC48972100,AC49178100,AC49755100,AC49904100,AC50177100,AC54859100,AC55957100,AC57235100,AC57246100,AC57413100,AC58258100,AC59022100,BB25098100,BC19238100,BC19631100,BC19632100,BC22124100,BC22151100,BC22152100,BC23518100,BC23603100,BC24304100,BC24725100,BC24855100,A055915100,AA56318100,AA56320100,AA58268100,AB46315100,AB46661100,AB50240100,AB55028100,AB55557100,AB55585100,AB55931100,AB56670100,AB56697100,AB57103100,AB57178100,AB57204100,AB57234100,AB57343100,AB57415100,AB57864100,AC45203100,AC45847100,AC47911100,AC49610100,AC49739100,AC49835100,AC49887100,AC52436100,AC52593100,AC52614100,AC55296100,AC55531100,AC55558100,AC55950100,AC56689100,AC56745100,AC57232100,AC57318100,AC57342100,AC57380100,AC57381100,AC57410100,AC57821100,AC57909100,AC58085100,AC58090100,AC58169100,AC58170100,AC58190100,AC58191100,AC58199100,AC58231100,AC58235100,AC58273100,AC58537100,AC58596100,AC58641100,AC58768100,AC58834100,AC58841100,AC59032100,AC59196100,AC59197100,AC59199100,AC59214100,AC59260100,AC59278100,AC59407100,AC59736100,BA24634100,BA25589100,BB26409100,BB26577100,BC21914100,BC22551100,BC22843100,BC23128100,BC23161100,BC23162100,BC23373100,BC23374100,BC23655100,BC24497100,BC24645100,BC24655100,BC25005100,BC25095100,BC25197100,BC25210100,BC25342100,BC25756100,BC25766100,BC25879100,BC25897100,BC25965100,BC26321100,BC26322100,BC26369100,BC26391100,BC26446100,BC26463100,BC26464100,BC26472100,BC26503100,BC26557100,BC26659100,BC26820100,BC26830100,BC26967100,BC26992100,BC27253100,BC27433100,BC27440100,BC27441100,AC58370100,BC24823100,BC26467100,BC26468100,BC25492100,BC25493100,BC25494100,BC25495100,BC25496100,BC26670100,BC26671100,BC26672100,A056719100,AA48757100,AB50431100,AB57116100,AB57237100,AC48990100,AC52568100,AC57153100,AC57371100,AC58064100,AC59742100,AC59809100,AC59841100,BC22989100,BC23109100,BC23220100,BC23266100,BC23267100,BC23649100,BC23654100,BC23922100,BC24521100,BC24592100,BC24643100,BC24646100,BC24968100,BC24969100,BC25207100,BC25208100,BC25501100,BC26048100,BC26209100,BC26210100,BC26398100,BC26399100,BC26400100,BC26496100,BC26537100,BC27014100 |
| Anti-diabetic agents | K000663299,K000739299,KC00595266,KC00596266,KC00663209,KC00729266,KC00739209,KC00795266,KC00803266,KC00823266,KC00898266,KC00899266,KC00900266,K000657299,K000760299,KC00657209,KC00760209,K000653299,KC00653209,KC00820266,KC00908266,KC00728266,KC00745209,KC00810266,KC00986266,KC01011272,KC01054266,KC01080216,A035324100,A0353241G0,A036349100,A038498100,A0384981G0,A039546100,A042464100,A0424641G0,A046686100,A047089100,A0488681G0,A050119100,AB34096100,AB340961G0,AB35665100,AB356651G0,AB408351G0,AB41233100,AB412331G0,AB43706100,AB437061G0,AB47532100,AB475321G0,AB47991100,AB48130100,AB48229100,AB48484100,AB484841G0,AB49457100,AB494571G0,AB49599100,AB52485100,AB524851G0,AB55258100,AB552581G0,AB55298100,AB56661100,AB56756100,AB567561G0,AB57217100,AB572171G0,AB58280100,AB582801G0,AC10323100,AC36201100,AC362011G0,AC36204100,AC362041G0,AC363491G0,AC36820100,AC368201G0,AC38680100,AC386801G0,AC40047100,AC400471G0,AC40098100,AC40119100,AC401191G0,AC40781100,AC407811G0,AC407811G4,AC407811G7,AC40835100,AC417501G0,AC42916100,AC429161G0,AC44233100,AC47086100,AC470861G0,AC47594100,AC47687100,AC479911G0,AC481301G0,AC482291G0,AC48734100,AC48863100,AC488631G0,AC48868100,AC48910100,AC49531100,AC495991G0,AC49659100,AC49957100,AC50080100,AC52534100,AC525341G0,AC552981G0,AC55985100,AC559851G0,AC56652100,AC566611G0,AC57171100,AC571711G0,AC57172100,AC571721G0,AC57177100,AC571771G0,AC57244100,AC57265100,AC57979100,AC58072100,AC58257100,AC58523100,AC585231G0,AC58534100,AC585341G0,AC58564100,AC585641G0,AC58613100,AC586131G0,AC589741G0,AC59009100,AC59654100,AC596541G0,AC59686100,AC596861G0,BA25308100,BA253081G0,BC071521G0,BC182311G0,BC22662100,BC226621G0,BC22663100,BC241891G0,BC25182100,BC25635100,BC26637100,BC271571G0,BC274021G0,BC274151G0,A003792100,A006823100,A022634100,A029325100,A029824100,A030512100,A0345501G0,A035254100,A035414100,A0354141G0,A036917100,A038681100,A040641100,A0406411G0,A042395100,A043051100,A043520100,A043858100,AB30970100,AB34736100,AB347361G0,AB40583100,AB405831G0,AB42908100,AB429081G0,AB43265100,AB432651G0,AB46074100,AB46658100,AB46766100,AB47070100,AB470701G0,AB47172100,AB48089100,AB49225100,AB49466100,AB55560100,AB57845100,AC02863100,AC028631G0,AC04715100,AC047151G0,AC20713100,AC207131G0,AC216401G0,AC226341G0,AC28245100,AC282451G0,AC29337100,AC293371G0,AC29513100,AC295131G0,AC30305100,AC303051G0,AC30698100,AC306981G0,AC309701G0,AC32831100,AC34348100,AC343481G0,AC34550100,AC34621100,AC346211G0,AC34893100,AC35670100,AC35795100,AC357951G0,AC35806100,AC36240100,AC362401G0,AC36395100,AC363951G0,AC36431100,AC37527100,AC375271G0,AC38181100,AC38500100,AC39144100,AC39408100,AC394081G0,AC39734100,AC397341G0,AC40233100,AC402331G0,AC41563100,AC41597100,AC41805100,AC42888100,AC43177100,AC435201G0,AC44172100,AC441721G0,AC44419100,AC45703100,AC46070100,AC46423100,AC464231G0,AC46640100,AC46647100,AC47049100,AC47231100,AC472311G0,AC47258100,AC47485100,AC47790100,AC47871100,AC48068100,AC480891G0,AC48121100,AC48307100,AC483071G0,AC48510100,AC485101G0,AC48560100,AC48846100,AC49072100,AC49114100,AC491141G0,AC492251G0,AC494661G0,AC50091100,AC50181100,AC501811G0,AC50769100,AC507691G0,AC52113100,AC54857100,AC55269100,AC57117100,AC57168100,AC57921100,AC579211G0,AC57989100,AC58088100,AC58121100,AC59396100,AC59625100,BB25266100,BC12873100,BC128731G0,BC224421G0,BC22671100,BC235031G0,A047680100,AB47671100,AB47811100,AB47848100,AB47981100,AB47984100,AB51714100,AB57312100,AC45646100,AC46488100,AC47307100,AC47878100,AC48095100,AC48228100,AC48855100,AC48898100,AC49204100,AC49494100,AC49579100,AC49697100,AC49829100,AC50084100,AC57326100,AC58092100,AC58241100,AC58388100,AC58975100,AC58976100,AC58977100,BB20786100,BB20787100,A049615100,A052589100,AA48333100,AA49116100,AA49429100,AA49500100,AA49930100,AA57769100,AB49581100,AB55275100,AC48007100,AC48057100,AC48098100,AC48516100,AC48602100,AC48753100,AC49085100,AC49119100,AC49549100,AC49560100,AC49625100,AC50043100,AC50130100,AC50426100,AC55011100,AC56669100,AC56692100,AC57872100,AC57897100,AC58350100,AC58410100,BC23206100,BC23207100,A044073121,A049924121,AC47403121,A049144100,AA49061100,AB46620100,AB49532100,AB52308100,AB57225100,AB57327100,AC47441100,AC48660100,AC49995100,AC58068100,BC22641100,BC26213100,BC26269100,AC47129100,AC47266100,AC47631100,AC47750100,AC50173100,BC23244100,BC23245100,AA52337100,BC25004213,BC25004297,KC00914216,BC27048263,BC27049263,KC00978206,KC00979206,BC26950100,BC26475100,BC26476100,BC26405100,BC26406100,BC27458100,BC26298100,BC26299100,BC26300100,BC25537100,BC25220100,BC25221100,AC58620100,AC59308100,AC60186100,BC24668100,AC59782100,BC25306100,A046732100,AB58071100,AC46733100,AC57799100,AC57860100,AC57861100,AC58954100,AC59300100,AC59393100,AC59759100,BA24876100,BC24005100,BC24006100,BC24839100,BC25041100,BC25043100,BC25453100,BC25454100,BC25455100,BC25480100,BC25481100,BC25482100,BC25792100,BC25793100,BC25794100,BC26681100,BC26682100,BC26683100,BC26685100,BC26686100,BC27035100,BC27036100,BC27037100,BC27038100,BC27039100,BC27040100,BC27073100,BC27074100,BC27114100,BC27115100,BC27116100,BC27117100 |
| Lipid-lowering agents | A055967100,AB47348100,AC44998100,AC46402100,AC47775100,AC47907100,AC47924100,AC47928100,AC48608100,AC48813100,AC48926100,AC49190100,AC49360100,AC49535100,AC49661100,AC49672100,AC49699100,AC49792100,AC49841100,AC49997100,AC52465100,AC52479100,AC56804100,AC56806100,AC57176100,AC58207100,BC23970100,BC24339100,BC24868100,BC25211100,A042389100,AC39307100,AC39403100,AC39601100,AC42539100,AC42558100,AC42627100,AC43573100,B025412100,A043887100,A046022100,AB46029100,AB48586100,AB48644100,AB48681100,AB49021100,AB49143100,AB49454100,AB49503100,AC47341100,AC48469100,AC48513100,AC48684100,AC51523100,AC52581100,AC57126100,AC57741100,B024297100,BC23596100,BC23597100,AC56629100,BC21198100,BC21199100,BC23556100,BC26147100,AA48879100,AA49226100,AA49288100,AA49543100,AA56739100,AA57774100,AA57930100,AA57950100,AB51732100,AB54967100,AB57772100,AB57967100,AB58049100,AC50086100,AC51598100,AC52301100,AC52530100,AC55268100,AC55272100,AC55583100,AC55895100,AC55952100,AC55956100,AC56319100,AC56682100,AC56791100,AC57133100,AC57267100,AC57805100,AC58041100,AC58211100,AC58366100,AC58401100,AC58579100,BA25200100,BA25201100,BA25337100,BC22886100,BC22889100,BC22890100,BC26028100,BC26350100,BC27256100,BC27339100,AA57802100,AA57843100,AA57880100,AA58282100,AB57194100,AB57940100,AC57130100,AC57803100,AC57809100,AC58067100,AC58098100,AC58270100,AC58291100,AC58315100,AC58316100,AC58384100,AC58396100,AC58411100,AC58605100,AC58621100,AC58622100,AC58813100,AC58822100,AC59240100,AC59265100,AC59266100,AC59649100,AC59652100,AC60114100,AC60197100,BA25797100,BA25798100,BA26332100,BA26504100,BC24129100,BC24131100,BC24597100,BC25796100,BC26226100,BC26367100,BC26368100,BC26497100,BC26505100,BC26543100,BC26544100,BC26900100,BC27044100,AA57372100,AA58648100,AC58078100,AC58525100,AC58526100,AC58633100,AC58639100,AC59192100,AC59193100,AC59398100,AC60174100,AC60175100,BC25350100,BC27002100,A006865100,A027676100,A030590100,A036374100,A038102100,A042585100,AB31138100,AB38016100,AB41332100,AB413321G0,AB47228100,AB49551100,AC23998100,AC276761G0,AC29811100,AC30766100,AC31284100,AC31609100,AC31641100,AC31807100,AC31954100,AC32830100,AC32833100,AC328331G0AC32985100,AC33476100,AC33733100,AC33934100,AC35439100,AC35838100,AC358381G0,AC36483100,AC364831G0,AC37685100,AC38296100,AC39553100,AC39731100,AC41496100,AC41837100,AC41931100,AC419311G0,AC42244100,AC42619100,AC42775100,AC42826100,AC42990100,AC44461100,AC45374100,AC49333100,AC49808100,AC50087100,AC55927100,AC56720100,AC56754100,AC57730100,AC57944100,AC59363100,BC07125100,BC16094100,BC22654100,BC23215100,BC23770100,BC25594100,A036423127,AC35424127,AC28958100,BC17140100,A027087100,AC27449100,B020276100,BC24058100,BC26552100,BC27311100,YC00018209,AC57216100,AC59251100,BC24250100,BC26169100,BC26643100,BC27283100,BC27534100,BC27535100 |

**Table S2.1 The range of combined lipid change in different lipid profiles**

| Variable (mg/dL) | Total cholesterol | Triglycerides | LDL cholesterol | NonHDL cholesterol | Apolipoprotein B |
| --- | --- | --- | --- | --- | --- |
| Low risk group |  |  |  |  |  |
| Baseline lipid level | 82.0-180.0 | 28.0-102.0 | 38.0-113.0 | 55.0-124.0 | 22.0-86.0 |
| Lipid level change |  |  |  |  |  |
| Decreasing or stable | -111.0-9.0 | -63.0-20.0 | -72.0-6.0 | -98.8-8.7 | -44.0-6.0 |
| Increasing | 9.0-157.0 | 21.0-366.0 | 6.0-110.0 | 8.7-107.6 | 6.0-65.0 |
| High risk group |  |  |  |  |  |
| Baseline lipid level | 180.0-660.0 | 103.0-774.0 | 113.0-252.0 | 124.0-631.0 | 86.0-199.0 |
| Lipid level change |  |  |  |  |  |
| Decreasing or stable | -265.0-9.0 | -600.0-20.0 | -150.0-6.0 | -247.2-8.7 | -93.0-6.0 |
| Increasing | 9.0-109.0 | 21.0-968.0 | 6.0-116.0 | 8.7-130.4 | 6.0-59.0 |

**Table S2.2 The median value of combined lipid group in different lipid profiles**

| mg/dL | Median value | Low risk group | Low risk group | High risk group | High risk group |
| --- | --- | --- | --- | --- | --- |
|  |  | decreasing or stable | increasing | decreasing or stable | increasing |
| Total cholesterol | 186 | 162 | 157 | 210 | 200 |
| LDL cholesterol | 117 | 98 | 96 | 134 | 128 |
| NonHDL cholesterol | 130 | 107 | 104 | 152 | 141 |
| Apolipoprotein B | 90 | 74 | 70 | 107 | 99 |

**Table S3 Correlation coefficients between various lipid profiles**

| Lipid | TG | LDL-C | NonHDL-C | ApoB | ∆TC | ∆TG | ∆LDL-C | ∆NonHDL-C | ∆ApoB |
| --- | --- | --- | --- | --- | --- | --- | --- | --- | --- |
| TC | 0.33 ^*^ | 0.88 ^*^ | 0.91 ^*^ | 0.78 ^*^ | 0.05 ^*^ | 0.01 | 0.11 ^*^ | 0.14 ^*^ | -0.01 ^*^ |
| TG |  | 0.30 ^*^ | 0.55 ^*^ | 0.41 ^*^ | -0.04 ^*^ | 0.04 ^*^ | -0.18 ^*^ | -0.13 ^*^ | -0.11 ^*^ |
| LDL-C |  |  | 0.87 ^*^ | 0.81 ^*^ | 0.05 ^*^ | -0.04 | 0.25 ^*^ | 0.15 ^*^ | -0.07 ^*^ |
| NonHDL-C |  |  |  | 0.80 ^*^ | 0.04 ^*^ | 0.00 | 0.10 ^*^ | 0.08 ^*^ | -0.10 ^*^ |
| ApoB |  |  |  |  | 0.05 ^*^ | 0.02 ^*^ | 0.20 ^*^ | 0.14 ^*^ | -0.21^*^ |
| ∆TC |  |  |  |  |  | 0.26 ^*^ | 0.76 ^*^ | 0.90 ^*^ | 0.70^*^ |
| ∆TG |  |  |  |  |  |  | 0.04 ^*^ | 0.36 ^*^ | 0.21 ^*^ |
| ∆LDL-C |  |  |  |  |  |  |  | 0.82 ^*^ | 0.63 ^*^ |
| ∆NonHDL-C |  |  |  |  |  |  |  |  | 0.68 ^*^ |

The correlation coefficients between baseline lipid level was derived by Pearson correlation; the correlation coefficients between baseline and the change of lipid level was calibrated by Oldham’s method; ^*^ *p*< 0.05. Abbreviations: TC, total cholesterol; TG, triglycerides; LDL-C, low density lipoprotein cholesterol; NonHDL-C, non-high density lipoprotein cholesterol; ApoB, apolipoprotein B.

**Table S4 Hazard ratios (and 95% CI values) of all-cause death risk during a median 13.4-year follow-up according to baseline lipid profiles in TwSHHH 2002**

|  |  | 1 | 2 | 3 | 4 | Trend test |
| --- | --- | --- | --- | --- | --- | --- |
| Total cholesterol | Events | 57 | 78 | 96 | 114 |  |
|  | Person-years | 12804 | 14444 | 14377 | 14874 |  |
|  | Incidence rate | 4.5 | 5.4 | 6.7 | 7.7 |  |
|  | Model1 | 1 | 1.19(0.79-1.81) | 1.32(0.88-1.98) | 1.37(0.92-2.04) | 0.12 |
|  | Model2 | 1 | 1.19(0.79-1.82) | 1.30(0.87-1.96) | 1.27(0.85-1.90) | 0.29 |
|  | Model3 | 1 | 1.02(0.68-1.54) | 0.88(0.58-1.33) | 1.01(0.68-1.5) | 0.98 |
| Triglycerides | Events | 47 | 67 | 91 | 137 |  |
|  | Person-years | 13102 | 13632 | 14651 | 14732 |  |
|  | Incidence rate | 3.6 | 4.9 | 6.2 | 9.3 |  |
|  | Model1 | 1 | 1.14(0.72-1.81) | 1.26(0.81-1.95) | 1.91(1.26-2.89) | 0.000 |
|  | Model2 | 1 | 1.08(0.68-1.72) | 1.15(0.73-1.80) | 1.67(1.09-2.57) | 0.002 |
|  | Model3 | 1 | 0.77(0.48-1.22) | 0.99(0.64-1.53) | 1.15(0.75-1.78) | 0.09 |
| LDL cholesterol | Events | 56 | 74 | 100 | 115 |  |
|  | Person-years | 12555 | 14508 | 14866 | 14571 |  |
|  | Incidence rate | 4.5 | 5.1 | 6.7 | 7.9 |  |
|  | Model1 | 1 | 0.87(0.57-1.35) | 1.24(0.84-1.83) | 1.21(0.82-1.78) | 0.14 |
|  | Model2 | 1 | 0.84(0.54-1.29) | 1.17(0.79-1.74) | 1.10(0.74-1.64) | 0.32 |
|  | Model3 | 1 | 0.72(0.47-1.10) | 0.82(0.55-1.22) | 0.86(0.58-1.27) | 0.86 |
| NonHDL cholesterol | Events | 51 | 70 | 94 | 130 |  |
|  | Person-years | 12601 | 14074 | 15047 | 14778 |  |
|  | Incidence rate | 4 | 5 | 6.2 | 8.8 |  |
|  | Model1 | 1 | 0.99(0.64-1.55) | 1.22(0.80-1.85) | 1.46(0.98-2.19) | 0.016 |
|  | Model2 | 1 | 0.93(0.60-1.46) | 1.12(0.73-1.72) | 1.30(0.86-1.96) | 0.07 |
|  | Model3 | 1 | 0.78(0.50-1.22) | 0.80(0.52-1.22) | 0.93(0.62-1.40) | 0.76 |
| Apolipoprotein B | Events | 41 | 71 | 103 | 130 |  |
|  | Person-years | 12205 | 14451 | 14645 | 15156 |  |
|  | Incidence rate | 3.4 | 4.9 | 7 | 8.6 |  |
|  | Model1 | 1 | 1.20(0.74-1.95) | 1.52(0.96-2.41) | 1.72(1.10-2.70) | 0.006 |
|  | Model2 | 1 | 1.10(0.67-1.79) | 1.39(0.87-2.22) | 1.45(0.91-2.31) | 0.06 |
|  | Model3 | 1 | 0.96(0.59-1.54) | 1.01(0.63-1.62) | 1.10(0.69-1.75) | 0.48 |

Incidence rates are presented per 1,000 person-years; Model 1: adjusted for age groups (20-64/≥65 years old) and sex. Model 2: as for model 1 plus body mass index (18.4/18.5-23.9/24.0–26.9/≥27 kg/m2), current smoker (yes/no), alcohol drinking (yes/no), marital status (single, divorced or separated/Living with spouse), regular exercise habit (yes/no),education level (9 years/at least 9 years), income level (monthly income level <40000,≥40000 New Taiwan Dollars). Model 3: as for model 2 plus baseline hypertension (yes/no), diabetes mellitus (yes/no), menopause(yes/no), family history of CVD (yes/no), and hsCRP level

**Table S5.1** **Subgroup analyses for the associations of the levels of the lipoproteins (higher risk group vs. lower risk group) with incident coronary heart disease**

| Variable | Total cholesterol | *p***_interaction_** | Triglycerides | *p***_interaction_** | LDL cholesterol | *p***_interaction_** | NonHDL cholesterol | *p***_interaction_** | Apolipoprotein B | *p***_interaction_** |
| --- | --- | --- | --- | --- | --- | --- | --- | --- | --- | --- |
| Age(years) |  | 0.71 |  | 0.96 |  | 0.71 |  | 0.96 |  | 0.57 |
| 20-64 | 1.24(0.74-2.07) |  | 1.32(0.75-2.31) |  | 1.21(0.72-2.03) |  | 1.06(0.62-1.82) |  | 1.26(0.72-2.22) |  |
| ≥65 | 1.57(0.78-3.13) |  | 1.37(0.69-2.69) |  | 1.59(0.76-3.33) |  | 1.22(0.59-2.53) |  | 1.10(0.55-2.19) |  |
| Sex |  | 0.87 |  | 0.46 |  | 0.27 |  | 0.36 |  | 0.09 |
| Women | 1.33(0.63-2.80) |  | 1.65(0.78-3.48) |  | 1.01(0.50-2.04) |  | 0.89(0.44-1.83) |  | 0.79(0.40-1.56) |  |
| Men | 1.48(0.90-2.42) |  | 1.23(0.72-2.10) |  | 1.62(0.95-2.77) |  | 1.35(0.78-2.34) |  | 1.69(0.94-3.05) |  |
| Target achieved of LDL-C |  | 0.07 |  | 0.54 |  | 0.28 |  | 0.43 |  | 0.023 |
| Yes | 1.28(0.79-2.06) |  | 1.44(0.86-2.41) |  | 1.30(0.80-2.13) |  | 1.23(0.75-2.01) |  | 1.56(0.93-2.61) |  |
| No | 7.73(1.02-58.58) |  | 1.10(0.49-2.46) |  | 4.45(0.59-33.48) |  | 0.85(0.19-3.77) |  | 0.41(0.16-1.04) |  |
| hsCRP level |  | 0.31 |  | 0.27 |  | 0.63 |  | 0.74 |  | 0.28 |
| hsCRP < median | 1.87(0.90-3.88) |  | 1.71(0.81-3.59) |  | 1.21(0.61-2.40) |  | 1.22(0.58-2.55) |  | 1.63(0.75-3.51) |  |
| hsCRP ≥ median | 1.31(0.80-2.15) |  | 1.19(0.70-2.02) |  | 1.57(0.91-2.72) |  | 1.12(0.66-1.92) |  | 1.07(0.63-1.82) |  |

**Table S5.2** **Subgroup analyses for the associations of the levels of the lipoproteins (higher risk group vs. lower risk group) with incident ischemic stroke**

| Variable | Total cholesterol | *p***_interaction_** | Triglycerides | *p***_interaction_** | LDL cholesterol | *p***_interaction_** | NonHDL cholesterol | *p***_interaction_** | Apolipoprotein B | *p***_interaction_** |
| --- | --- | --- | --- | --- | --- | --- | --- | --- | --- | --- |
| Age(years) |  | 0.54 |  | 0.33 |  | 0.015 |  | 0.009 |  | 0.65 |
| 20-64 | 1.21(0.63-2.32) |  | 1.33(0.66-2.71) |  | 1.57(0.80-3.10) |  | 2.22(1.01-4.88) |  | 1.14(0.57-2.28) |  |
| ≥65 | 1.54(0.75-3.15) |  | 1.15(0.56-2.33) |  | 0.76(0.39-1.51) |  | 1.00(0.49-2.01) |  | 1.58(0.74-3.38) |  |
| Sex |  | 0.51 |  | 0.48 |  | 0.54 |  | 0.82 |  | 0.71 |
| Women | 1.17(0.56-2.42) |  | 1.57(0.74-3.32) |  | 0.99(0.48-2.02) |  | 1.48(0.68-3.23) |  | 1.50(0.68-3.27) |  |
| Men | 1.64(0.85-3.17) |  | 1.07(0.54-2.14) |  | 1.25(0.65-2.40) |  | 1.57(0.77-3.17) |  | 1.29(0.64-2.58) |  |
| Target achieved of LDL-C |  | 0.47 |  | 0.40 |  | 0.96 |  | 0.12 |  | 0.23 |
| Yes | 1.17(0.64-2.16) |  | 1.12(0.59-2.11) |  | 1.12(0.61-2.08) |  | 1.33(0.72-2.44) |  | 1.27(0.67-2.38) |  |
| No | 1.28(0.15-11.27) |  | 1.71(0.48-6.11) |  | 0.58(0.06-5.82) |  | 0.81(0.08-8.69) |  | 1.37(0.17-10.94) |  |
| hsCRP level |  | 0.033 |  | 0.13 |  | 0.27 |  | 0.09 |  | 0.10 |
| hsCRP < median | 0.53(0.22-1.26) |  | 0.47(0.20-1.14) |  | 0.64(0.27-1.49) |  | 0.73(0.31-1.73) |  | 0.65(0.28-1.51) |  |
| hsCRP ≥ median | 1.92(1.02-3.63) |  | 1.93(0.95-3.93) |  | 1.35(0.73-2.49) |  | 2.29(1.12-4.68) |  | 2.22(1.09-4.52) |  |

**Table S5.3** **Subgroup analyses for the associations of the levels of the lipoproteins (higher risk group vs. lower risk group) with all-cause death**

| Variable | Total cholesterol | *p***_interaction_** | Triglycerides | *p***_interaction_** | LDL cholesterol | *p***_interaction_** | NonHDL cholesterol | *p***_interaction_** | Apolipoprotein B | *p***_interaction_** |
| --- | --- | --- | --- | --- | --- | --- | --- | --- | --- | --- |
| Age(years) |  | 0.08 |  | 0.92 |  | 0.47 |  | 0.73 |  | 1.00 |
| 20-64 | 1.00(0.68-1.48) |  | 1.04(0.69-1.58) |  | 0.97(0.66-1.43) |  | 0.87(0.58-1.30) |  | 0.87(0.58-1.30) |  |
| ≥65 | 0.84(0.58-1.23) |  | 1.49(0.98-2.26) |  | 1.02(0.69-1.52) |  | 1.11(0.74-1.66) |  | 1.23(0.82-1.84) |  |
| Sex |  | 0.29 |  | 0.44 |  | 0.30 |  | 0.14 |  | 0.24 |
| Women | 0.78(0.49-1.22) |  | 1.10(0.69-1.75) |  | 0.93(0.59-1.46) |  | 0.84(0.53-1.33) |  | 0.88(0.56-1.39) |  |
| Men | 1.02(0.72-1.43) |  | 1.49(1.02-2.19) |  | 1.08(0.76-1.54) |  | 1.16(0.80-1.68) |  | 1.21(0.83-1.76) |  |
| Target achieved of LDL-C |  | 0.73 |  | 0.83 |  | 0.68 |  | 0.69 |  | 0.67 |
| Yes | 0.81(0.57-1.14) |  | 1.22(0.86-1.74) |  | 0.99(0.69-1.40) |  | 0.93(0.66-1.31) |  | 1.08(0.76-1.54) |  |
| No | 0.97(0.45-2.09) |  | 1.39(0.79-2.46) |  | 0.79(0.37-1.71) |  | 1.17(0.41-3.41) |  | 0.89(0.44-1.82) |  |
| hsCRP level |  | 0.71 |  | 0.58 |  | 0.92 |  | 1.00 |  | 0.80 |
| hsCRP < median | 0.67(0.41-1.09) |  | 1.00(0.61-1.64) |  | 0.76(0.46-1.23) |  | 0.83(0.51-1.38) |  | 0.73(0.44-1.21) |  |
| hsCRP ≥ median | 1.04(0.75-1.45) |  | 1.30(0.90-1.88) |  | 1.18(0.84-1.65) |  | 1.14(0.80-1.62) |  | 1.26(0.88-1.80) |  |

**Table S6.1 Sensitivity analyses for the associations of the levels of the lipoproteins specified by quartiles with incident cardiovascular disease**

|  | Q1 | Q2 | Q3 | Q4 |
| --- | --- | --- | --- | --- |
| Excluding events in the first year |  |  |  |  |
| Total cholesterol | 1 | 1.61(0.89-2.90) | 1.61(0.90-2.87) | 1.90(1.09-3.32) |
| Triglycerides | 1 | 1.06(0.58-1.95) | 1.39(0.79-2.44) | 1.33(0.75-2.36) |
| LDL cholesterol | 1 | 1.27(0.70-2.31) | 1.44(0.82-2.51) | 1.60(0.92-2.77) |
| NonHDL cholesterol | 1 | 1.66(0.87-3.17) | 1.60(0.86-2.98) | 1.83(0.99-3.39) |
| Apolipoprotein B | 1 | 1.92(0.92-4.02) | 1.92(0.92-3.97) | 2.29(1.11-4.69) |
| Excluding Triglyceride ≥ 400 mg/dL |  |  |  |  |
| Total cholesterol | 1 | 1.53(0.86-2.72) | 1.54(0.88-2.71) | 1.77(1.02-3.05) |
| Triglycerides | 1 | 0.93(0.52-1.67) | 1.25(0.73-2.14) | 1.18(0.68-2.05) |
| LDL cholesterol | 1 | 1.34(0.75-2.41) | 1.38(0.79-2.42) | 1.49(0.86-2.58) |
| NonHDL cholesterol | 1 | 1.61(0.86-3.01) | 1.47(0.81-2.70) | 1.68(0.93-3.06) |
| Apolipoprotein B | 1 | 1.98(0.95-4.14) | 1.89(0.91-3.92) | 2.16(1.05-4.42) |

**Table S6.2 Sensitivity analyses for the associations of the levels of the lipoproteins specified by quartiles with all-cause death**

|  | Q1 | Q2 | Q3 | Q4 |
| --- | --- | --- | --- | --- |
| Excluding events in the first year |  |  |  |  |
| Total cholesterol | 1 | 1.09(0.72-1.65) | 0.82(0.53-1.25) | 1.00(0.67-1.50) |
| Triglycerides | 1 | 0.88(0.54-1.43) | 1.10(0.70-1.74) | 1.28(0.81-2.02) |
| LDL cholesterol | 1 | 0.73(0.47-1.13) | 0.80(0.53-1.21) | 0.84(0.56-1.26) |
| NonHDL cholesterol | 1 | 0.82(0.52-1.29) | 0.80(0.51-1.24) | 0.92(0.60-1.40) |
| Apolipoprotein B | 1 | 0.97(0.60-1.58) | 1.04(0.64-1.67) | 1.04(0.64-1.67) |
| Excluding Triglyceride ≥ 400 mg/dL |  |  |  |  |
| Total cholesterol | 1 | 1.03(0.67-1.56) | 0.86(0.56-1.31) | 1.06(0.71-1.58) |
| Triglycerides | 1 | 0.76(0.48-1.21) | 0.98(0.63-1.51) | 1.14(0.74-1.77) |
| LDL cholesterol | 1 | 0.73(0.47-1.12) | 0.82(0.54-1.24) | 0.89(0.59-1.32) |
| NonHDL cholesterol | 1 | 0.80(0.51-1.25) | 0.79(0.51-1.22) | 0.95(0.62-1.44) |
| Apolipoprotein B | 1 | 1.01(0.61-1.66) | 1.12(0.69-1.82) | 1.19(0.73-1.93) |

**Table S7.1** **Sensitivity analyses for the associations of the combined lipid change with incident cardiovascular disease**

|  | Low risk group | | High risk group | |
| --- | --- | --- | --- | --- |
|  | Decreasing or Stable | Increasing | Decreasing or Stable | Increasing |
| New definition of stable lipid change^a^ |  |  |  |  |
| Total cholesterol | 1 | 1.91(1.11-3.30) | 1.48(0.99-2.22) | 2.03(1.16-3.53) |
| Triglycerides | 1 | 0.87(0.41-1.87) | 1.08(0.74-1.58) | 2.35(1.44-3.84) |
| LDL cholesterol | 1 | 1.29(0.74-2.25) | 1.13(0.75-1.71) | 1.94(1.19-3.17) |
| NonHDL cholesterol | 1 | 1.20(0.67-2.15) | 1.08(0.71-1.66) | 1.82(1.11-2.99) |
| Apolipoprotein B | 1 | 0.64(0.34-1.21) | 0.99(0.66-1.48) | 1.48(0.86-2.53) |
| Excluding events in the first year |  |  |  |  |
| Total cholesterol | 1 | 1.32(0.76-2.28) | 1.39(0.90-2.17) | 1.71(1.01-2.88) |
| Triglycerides | 1 | 1.07(0.59-1.95) | 1.19(0.78-1.84) | 1.83(1.11-3.02) |
| LDL cholesterol | 1 | 1.42(0.81-2.51) | 1.31(0.81-2.11) | 2.17(1.30-3.62) |
| NonHDL cholesterol | 1 | 1.27(0.71-2.28) | 1.24(0.76-2.03) | 1.69(0.99-2.87) |
| Apolipoprotein B | 1 | 0.70(0.38-1.26) | 1.07(0.67-1.70) | 1.09(0.63-1.89) |
| Excluding Triglyceride ≥ 400 mg/dL |  |  |  |  |
| Total cholesterol | 1 | 1.44(0.84-2.46) | 1.41(0.91-2.20) | 1.72(1.02-2.90) |
| Triglycerides | 1 | 1.07(0.60-1.93) | 1.17(0.77-1.79) | 1.77(1.08-2.90) |
| LDL cholesterol | 1 | 1.35(0.78-2.35) | 1.15(0.72-1.83) | 1.95(1.18-3.22) |
| NonHDL cholesterol | 1 | 1.32(0.75-2.31) | 1.17(0.72-1.90) | 1.63(0.97-2.74) |
| Apolipoprotein B | 1 | 0.78(0.43-1.39) | 1.06(0.67-1.69) | 1.04(0.60-1.80) |

^a^ ±0.5 baseline lipid standard deviation

**Table S7.2** **Sensitivity analyses for the associations of the combined lipid change with all-cause death**

|  | Low risk group | | High risk group | |
| --- | --- | --- | --- | --- |
|  | Decreasing or Stable | Increasing | Decreasing or Stable | Increasing |
| New definition of stable lipid change^a^ |  |  |  |  |
| Total cholesterol | 1 | 0.54(0.32-0.94) | 0.85(0.63-1.15) | 0.63(0.36-1.11) |
| Triglycerides | 1 | 1.71(1.02-2.87) | 1.37(0.98-1.90) | 1.81(1.13-2.90) |
| LDL cholesterol | 1 | 0.55(0.32-0.94) | 0.94(0.70-1.28) | 0.61(0.36-1.02) |
| NonHDL cholesterol | 1 | 0.57(0.34-0.98) | 0.91(0.66-1.26) | 0.72(0.45-1.16) |
| Apolipoprotein B | 1 | 0.54(0.32-0.90) | 0.88(0.64-1.22) | 0.97(0.60-1.57) |
| Excluding events in the first year |  |  |  |  |
| Total cholesterol | 1 | 0.37(0.23-0.61) | 0.70(0.51-0.95) | 0.45(0.27-0.74) |
| Triglycerides | 1 | 1.10(0.67-1.79) | 1.30(0.91-1.88) | 1.47(0.92-2.34) |
| LDL cholesterol | 1 | 0.54(0.33-0.88) | 0.93(0.67-1.29) | 0.46(0.28-0.76) |
| NonHDL cholesterol | 1 | 0.56(0.34-0.91) | 0.89(0.63-1.24) | 0.53(0.33-0.85) |
| Apolipoprotein B | 1 | 0.57(0.36-0.90) | 0.85(0.60-1.22) | 0.69(0.43-1.10) |
| Excluding Triglyceride ≥ 400 mg/dL |  |  |  |  |
| Total cholesterol | 1 | 0.41(0.25-0.67) | 0.79(0.58-1.08) | 0.47(0.28-0.77) |
| Triglycerides | 1 | 1.17(0.73-1.87) | 1.33(0.93-1.90) | 1.36(0.85-2.17) |
| LDL cholesterol | 1 | 0.51(0.31-0.83) | 0.94(0.68-1.30) | 0.51(0.32-0.83) |
| NonHDL cholesterol | 1 | 0.56(0.35-0.90) | 0.92(0.65-1.29) | 0.53(0.33-0.85) |
| Apolipoprotein B | 1 | 0.59(0.37-0.94) | 0.95(0.66-1.36) | 0.73(0.45-1.17) |

^a^ ±0.5 baseline lipid standard deviation
